# Supplementary figures and images for: Immunogenicity Persistence of Different Immunization Regimens of Rabies Vaccine in the 10–60 Years Age Group: A Follow-Up Report Based on Phase III Clinical Trial
Source: Vaccines (Basel). 2024 Oct 24;12(11):1209. doi: 10.3390/vaccines12111209 (PMC11598175; doi:10.3390/vaccines12111209)

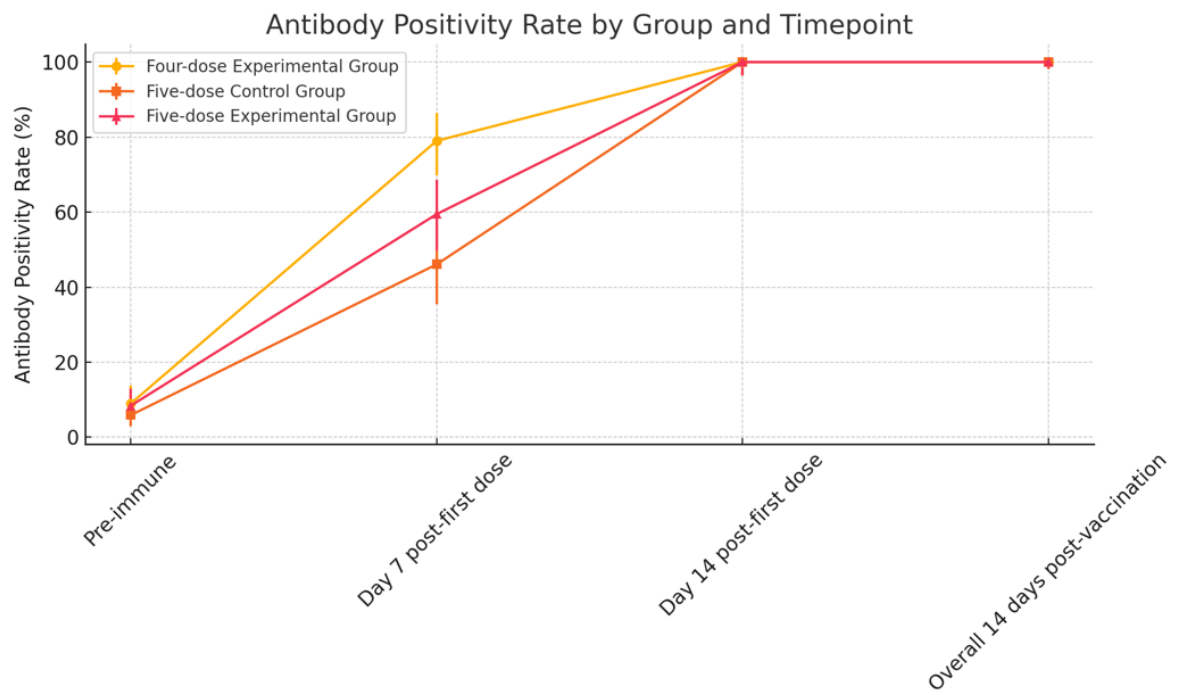

Supplementary Figure S1A

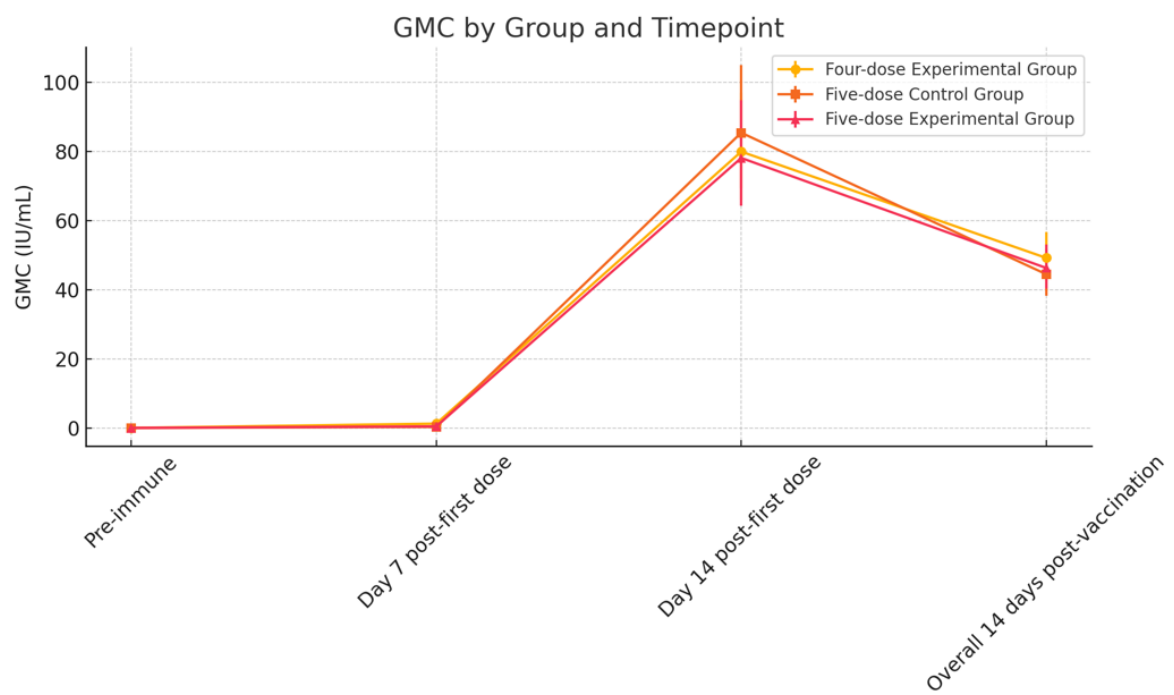

Supplementary Figure S1B

Supplement: Supplementary file 1 [file vaccines-12-01209-s001.zip › vaccines-3192861-supplementary.pdf]
